# Characterization and assessment of potential environmental risk of tailings stored in seven impoundments in the Aries river basin, Western Romania

**DOI:** 10.1186/1752-153X-7-5

**Published:** 2013-01-14

**Authors:** Erika Levei, Tiberiu Frentiu, Michaela Ponta, Claudiu Tanaselia, Gheorghe Borodi

**Affiliations:** 1 INCDO-INOE 2000, Research Institute for Analytical Instrumentation, 67 Donath, 400293, Cluj-Napoca, Romania; 2 Faculty of Chemistry and Chemical Engineering, Babes-Bolyai University, 11 Arany Janos, 400028, Cluj-Napoca, Romania; 3 National Institute for Research and Development of Isotopic and Molecular Technologies, 65-103 Donath, 400293, Cluj-Napoca, Romania

**Keywords:** Tailings, Hazardous/priority hazardous metal, Acid rock drainage, Environmental risk, Multivariate statistics

## Abstract

**Background:**

The objective of this study was to examine the potential environmental risk of tailings resulted after precious and base metal ores processing, stored in seven impoundments located in the Aries river basin, Romania. The tailings were characterized by mineralogical and elemental composition, contamination indices, acid rock drainage generation potential and water leachability of hazardous/priority hazardous metals and ions. Multivariate statistical methods were used for data interpretation.

**Results:**

Tailings were found to be highly contaminated with several hazardous/priority hazardous metals (As, Cu, Cd, Pb), and pose potential contamination risk for soil, sediments, surface and groundwater. Two out of the seven studied impoundments does not satisfy the criteria required for inert wastes, shows acid rock drainage potential and thus can contaminate the surface and groundwater. Three impoundments were found to be highly contaminated with As, Pb and Cd, two with As and other two with Cu. The tailings impoundments were grouped based on the enrichment factor, geoaccumulation index, contamination factor and contamination degree of 7 hazardous/priority hazardous metals (As, Cd, Cr, Cu, Ni, Pb, Zn) considered typical for the studied tailings. Principal component analysis showed that 47% of the elemental variability was attributable to alkaline silicate rocks, 31% to acidic S-containing minerals, 12% to carbonate minerals and 5% to biogenic elements. Leachability of metals and ions was ascribed in proportion of 61% to silicates, 11% to acidic minerals and 6% to the organic matter. A variability of 18% was attributed to leachability of biogenic elements (Na, K, Cl^-^, NO_3_^-^) with no potential environmental risk. Pattern recognition by agglomerative hierarchical clustering emphasized the grouping of impoundments in agreement with their contamination degree and acid rock drainage generation potential.

**Conclusions:**

Tailings stored in the studied impoundments were found to be contaminated with some hazardous/ priority hazardous metals, fluoride and sulphate and thus presents different contamination risk for the environment. A long term monitoring program of these tailings impoundments and the expansion of the ecologization measures in the area is required.

## Background

Exploitation of mineral resources containing base and precious metals is one of the main activities that have contributed to the development of humanity. In the same time, mining has a negative reputation because of the large amounts of associated wastes discharged in tailings impoundments, which has caused damages to the surrounding mining areas by soil, water and air pollution [1]. The tailings exposed to weathering are important sources of Acid Rock Drainage (ARD), which contaminates the environment with metals, non-metals and sulphate anions as a result of the sulphide oxidation and leaching process [2-7].

Although mining activity stopped in certain area, the potential risk of environment contamination still exists through the vast quantities of waste stored in tailings impoundments. The ARD generated by tailings represents in case of accidents the main pollution source of natural watercourses, sometimes with cross-border effects. It can be mentioned the accidents that occurred in January 2000 in Baia Mare and March 2000 in Baia Borsa in northern Romania, when more than 200000 m^3^ of waste water containing metals and cyanide from two tailings impoundments reached the Somes and Viseu rivers and finally the Danube via the Tisa river [8,9]. As a consequence of such accidents, elemental composition and mineralogical characterization of mining waste in order to identify hazardous/priority hazardous metals and reactive sulphides is required for the assessment of potential environmental risk [10-16].

The aim of this work was to characterize and evaluate the potential environmental risk of tailings impoundments located in the Aries river basin, Alba County, the Apuseni Mountains, Romania, related to porphyry copper and precious metals mining. Several complementary approaches were considered for this purpose. The mineralogical and elemental (Al, Fe, Ca, K, Mg, Na, Mn, Ba, Zn, Pb, Cu, Ni, Cr, As, Cd, Co, Sr, Ti, V, W, Ag, Au, S) composition of tailings were determined and the ARD generation potential was assessed using Net Neutralization Potential (NNP). It was studied the leachability of hazardous (Ag, As, Ba, Co, Cr, Cu, Ti, V, Zn)/priority hazardous (Cd, Ni, Pb) metals and several ions (F^-^, Cl^-^, NO_3_^-^, SO_4_^2-^, NH_4_^+^). The term ”hazardous/priority hazardous metals” was used throughout this paper considering their toxicity and classification in these two groups according to [17]. The terms “heavy metal” and “potentially toxic metal” were avoided as they have ambiguous uses in environmental quality assessments [18]. Tailings contamination was characterized by several indices calculated for seven hazardous/priority hazardous metals (As, Cd, Cr, Cu, Ni, Pb, Zn). The selection of these metals was made in accordance with Hakanson [19], thus for the assessment of contamination risk the typical parameters for the studied area were considered and not all toxic substances. Generally, these are metals with similar occurrence and behaviour under comparable conditions. Although, Ag has a similar toxicity with that of Cr, it was not considered in the calculus of contamination indices because the statistical analysis revealed that precious metals have low influence in describing the variability of tailings composition. The contamination risk of tailings for soil, river sediment, river and groundwater was assessed by comparing the determined metal and ion contents with the corresponding limit values. The contribution of pollutants to variability of tailings composition was evaluated using Principal Component Analysis (PCA). The impoundments were grouped according to the contamination degree and ARD generation potential using Agglomerative Hierarchical Clustering (AHC).

## Results and discussion

### Site description

Romania has a long mining history of base (Cu, Cd, Pb, Zn) and precious metals, most centres and tailings impoundments being located in the north of the country (Maramures County) or in the west in the Apuseni Mountains (Alba and Hunedoara Counties). The southern part of the Apuseni Mountains is recognized for its Au and Ag deposit within the Golden Quadrangle as well as the Rosia Poieni porphyry copper deposit, which together led to the development of prosperous mining activities. Besides the economic importance, the Apuseni Mountains zone is known for the tourism potential represented by several natural reservations and caves [20].

**Figure 1:**
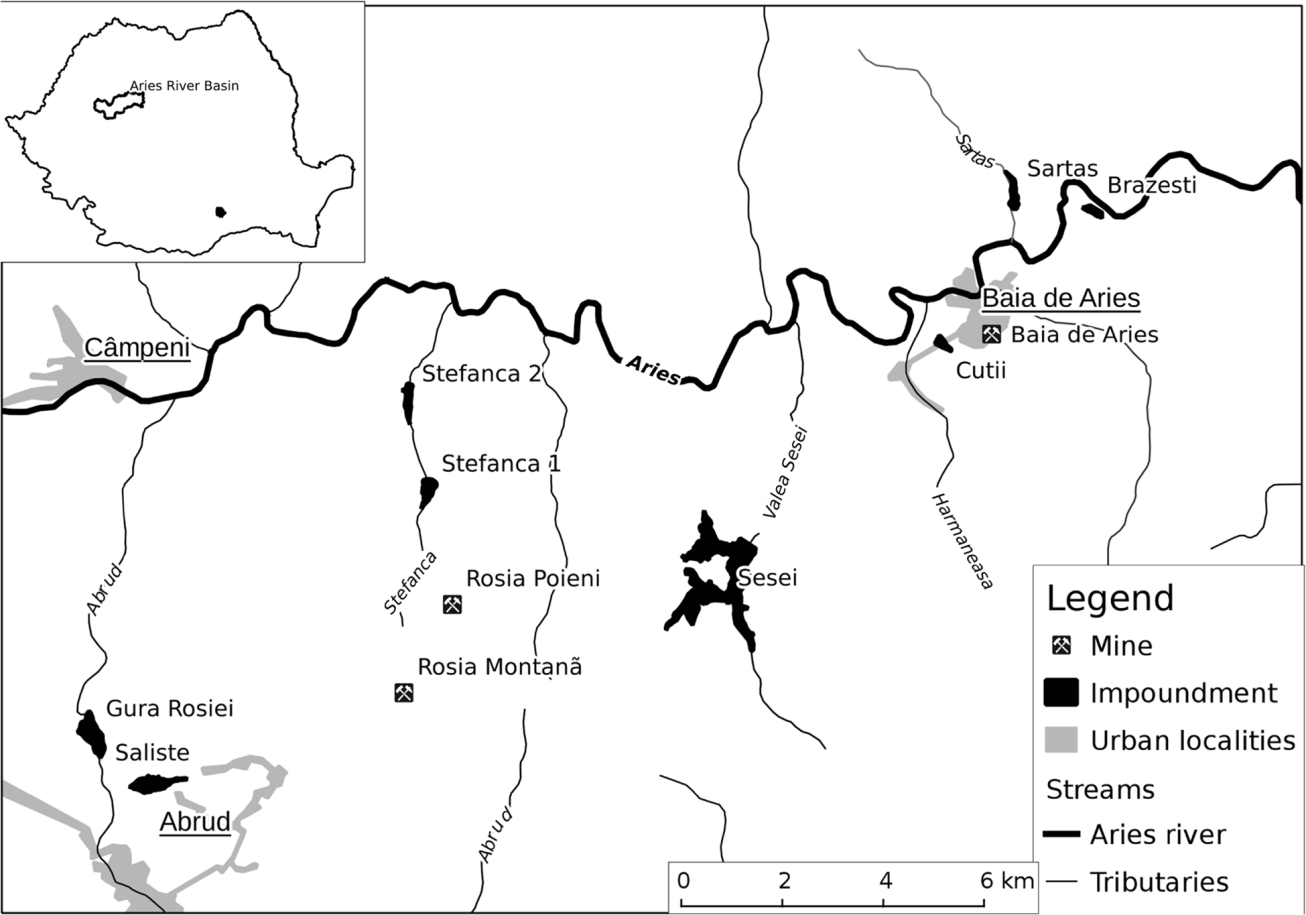
Location of tailings impoundments in the Aries river basin.

In the Aries river basin with an area of 3000 km^2^ in the Alba County there are several open pits and underground mines as well as the related tailings impoundments storing tailings from dressing of non-ferrous copper ores and precious metals. Seven tailings impoundments put into operation after 1963 with a surface of 444 ha and storing about 100 million m^3^ of tailings were considered in this study. The main characteristics of these impoundments and their locations are provided in Table 1 and Figure 1. Currently five of the seven are going green, whereas two (Stefanca and Sesei) are still in use for storing waste from the processing of the Rosia Poieni porphyry copper deposit mined in open pit [21].

**Table 1:** Characteristics of the tailings impoundments under study located in the Aries river basin

**Impoundment**	**River**	**Year of putting into operation**	**Current status**	**Type**	**Surface (ha)**	**Dam height (m)**	**Deposit volume (mil m**^**3**^**)**
Saliste	Abrud	1986	Closed since 2004, going green	Valley deposit	18.5	87	6.60
Gura Rosiei	Abrud	1967	Closed, going green	Slope deposit	23.0	44	5.80
Stefanca	Stefanca	1993	In operation (storage stopped)	Valley deposit	50.7	85	11.0
Sesei	Sesei	1985	In operation	Valley deposit	330	100	66.0
Cutii	Harmaneasa	1976	Closed since 2002, going green	Valley deposit	4.8	74	1.62
Sartas	Sartas	1993	Closed since 2005, going green	Valley deposit	6.0	78	2.86
Brazesti	Aries	1963	Going green	Slope deposit	10.7	42	3.47

There are few published data on the characterization and assessment of potential environmental risk of tailings in the area under study. Two papers should be mentioned, namely that of Sima et al. [3] who characterized the Mialu and Ribita tailings impoundments located in the Certej and Crisul Alb floodplains and that of Friedel et al. [22] about the influence of the Stefanca and Sesei impoundments on groundwater. The drainage, collection and leachate treatment systems of the tailings impoundments under study work improperly or are even missing in some cases. Consequently, they could pose potential risk of contamination for the environment in the Aries river basin.

### Mineralogical composition

Abundance of mineralogical species in tailings is summarized in Table 2. The mineralogical composition is dominated by primary minerals belonging to oxide (quartz) and aluminosilicate groups. The identified minor minerals were reactive sulphide and sulphate, which promote generation of ARD and carbonates with neutralization property. Traces of minerals containing As, Ba, Mn, Pb, Zn were also identified.

**Table 2:** Mineralogical composition of tailings

**Mineral group**	**Mineral species**	**Chemical formula**	**Impoundment**						
			**Saliste**	**Gura Rosiei**	**Stefanca**	**Sesei**	**Cutii**	**Sartas**	**Brazesti**
oxides	quartz	SiO_2_	++	++	++	++	++	+	++
silicates	albite	NaAlSi_3_O_8_	++		+	+			++
	muscovite	KAl2(Si_3_Al)O_10_(OH,F)_2_				+	+	++	+
	orthoclase	AlKSi_3_O_8_	++	++			+		
	biotite	NaFeKMgTiSi_3_O_12_		+	+		±	++	
	feldspar	Al_2_CaSr O_8_Si_2_			±				+
	anorthoclase	AlCaKNaO_8_Si_3_	±		++	++		+	
sulphides	chalcopyrite	CuFeS_2_			±		±	±	±
	pyrite	FeS_2_	±				±	±	
	pyrrhotite	Fe_9_S_10_		±			±		±
	sphalerite	ZnS						±	
	galena	PbS					±	±	±
sulphates	jarosite	KFe_3_(SO_4_)_2_(OH)_6_					±	+	
	iron sulphate	Fe_2_(SO_4_)_3_			+	+			
	gypsum	CaSO_4_					±	+	
	anglesite	PbSO_4_					±	±	
carbonates	eitelite	Na_2_Mg(CO_3_)_2_			±				+
	baritocalcite	BaCa(CO_3_)_2_			+	±			
	calcite	CaCO_3_			±	±			+
	rhodochrosite	MnCO3						±	
	dolomite	CaMg(CO_3_)_2_		+					+
arsenic minerals	wilhelmkleinite	As_2_Fe_2_Zn O_8_(OH)_2_		±	±				±
	potassium hydroxo pentafluoro arsenate	AsF_5_K(OH)							±

(++) - major (20–50%); (+) - minor (5–20%); (±) - trace (<5%).

### Elemental composition of tailings and leachability of metals and ions

The total and the water leachable metals and ions content at a solid-to-water ratio of 1:10 are presented in Table 3. Data reveal high differences from one impoundment to another in terms of total contents and water mobility of hazardous (As, Cu, Cr, Zn)/priority hazardous (Cd, Pb, Ni) metals as well as ions. The mobilization of the leaching fraction by weathering processes may pose a major environmental hazard.

**Table 3:** Total content of metals and the leached fraction of metals and ions by water

**Parameter**	**Impoundment**													
	**Saliste**		**Gura Rosiei**		**Stefanca**		**Sesei**		**Cutii**		**Sartas**		**Brazesti**	
	**Total**	**Leached**	**Total**	**Leached**	**Total**	**Leached**	**Total**	**Leached**	**Total**	**Leached**	**Total**	**Leached**	**Total**	**Leached**
	**mg kg**^**-1**^													
Al	1810	10.1	2670	2.25	15600	17.6	13000	4.25	1530	77.6	5580	217	3080	2.27
Fe	9350	10.4	4650	1.1	13300	*0.06*	15700	2.2	15800	403	15600	110	13800	*0.06*
Mn	55.0	1.7	83.0	0.93	482	0.03	589	0.15	84.0	13.6	285	75.0	420	0.11
Ba	103	0.06	47.5	0.07	120	0.27	144	0.240	64.2	0.120	114	0.160	72.1	0.090
Zn	185	4.6	46.0	0.60	115	0.08	109	0.520	204	14.2	401	45.2	330	0.160
Pb	117	0.049	115	0.051	25.4	0.009	31.3	0.080	528	0.520	649	0.540	551	0.043
Cu	15.9	0.563	4.8	0.094	579	0.037	850	0.355	107	4.89	133	9.19	65.7	0.021
Ni	2.0	0.165	1.0	0.044	3.4	0.05	1.8	0.044	1.8	0.48	11.6	1.57	5.2	0.059
Cr	2.5	0.166	1.0	0.021	4.1	0.015	3.4	0.057	2.4	0.283	9.8	0.300	4.3	0.050
As	101	0.031	50.8	0.034	5.5	0.021	7.4	0.137	483	0.560	575	0.530	655	0.600
Cd	0.60	0.050	0.10	0.012	0.20	0.01	0.40	0.013	0.40	0.080	1.5	0.351	1.6	0.010
Co	0.30	0.023	0.10	0,011	6.2	*0.001*	10.2	0.002	0.60	0.148	2.2	0.718	3.3	*0.001*
V	37.0	*0.002*^*a*^	13.8	*0.002*	96.8	*0.002*	179	*0.002*	11.2	*0.002*	12.8	*0.002*	7.7	*0.002*
Ag	10.5	*0.001*	8.0	*0.001*	0.30	*0.001*	0.50	*0.001*	5.3	*0.001*	5.8	*0.001*	2.2	*0.001*
F-	-	1.8	-	*0.15*	-	2.4	-	1.4	-	11.0	-	13.0	-	0.80
Cl^-^	-	9.3	-	5.3	-	25.0	-	0.400	-	17.0	-	9.3	-	3.4
SO_4_^2-^	-	700	-	46.4	-	1100	-	217	-	17000	-	17000	-	240
NO_3_^-^	-	*0.15*	-	0.8	-	1.9	-	*0.15*	-	*0.15*	-	*0.15*	-	*0.15*
NH_4_^+^	-	3.5	-	5.5	-	3.5	-	4.2	-	6.2	-	5.2	-	2.7

^a^ half of the detection limit.

### Enrichment factors and contamination degree with hazardous/priority hazardous metals

In order to assess the extent of tailings contamination the following indices were calculated for seven metals (Pb, Cu, Zn, As, Cd, Ni, Cr): enrichment factor (E_f_) according to Sinex and Helz [23], geoaccumulation index (I_geo_), introduced by Muller [24], and contamination factor (C_f_) and contamination degree (C_d_) according to Hakanson [19]. The C_d_ levels resulted by summing the C_f_ values of metals served to classify tailings on different contamination levels. For calculation of indices, as reference, was used the natural background levels of metals in the upper continental crust: Pb 20, Cu 25, Zn 71, As 1.5, Cd 0.098, Ni 20, Cr 35 and Fe 35000 mg kg^-1^ according to Taylor and McLeenan [25].

Contamination indices and ranking of tailings from the impoundments under study are given in Table 4. Although concentration of metals in tailings are too low to be today economically attractive for recovery purpose, some hazardous/priority hazardous metals exhibit an extremely severe enrichment (E_f_ increases in the order Cd < Pb < Cu < As) compared to the continental crust and can be important contamination sources for soil and sediment.

**Table 4:** **Contamination indices and ranking of tailings impoundments located in the Aries river basin**^**a**^

**Enrichment factor (E**_**f**_**)**							
**Impoundment**	**No (0 < E**_**f**_ **≤ 1)**	**Minor (1 < E**_**f**_ **≤ 3)**	**Moderate (3 < E**_**f**_ **≤ 5)**	**Moderately severe (5 < E**_**f**_ **≤ 10)**	**Severe (10 < E**_**f**_ **≤ 25)**	**Very severe (25 < E**_**f**_ **≤ 50)**	**Extremely severe (E**_**f**_ **> 50)**
Saliste	Ni < Cr	Cu	-	Zn(9.8)	Pb(22), Cd(23)	-	As(251)
Gura Rosiei	Cr < Ni	Cu	Zn	Cd(7.7)	-	Pb(43)	As(255)
Stefanca	Cr < Ni	-	Pb < Zn	Cd(5.4), As(9.6)	-	-	Cu(61)
Sesei	Ni < Cr	-	Pb < Zn	Cd(9.1)	As(11)	-	Cu(76)
Cutii	Ni < Cr	-	-	Zn(6.4), Cd(9.1), Cu(9.5)	-	-	Pb(59), As(715)
Sartas	Cr	Ni	-	-	Cu(12), Zn(13)	-	Cd(34), Pb(73), As(860)
Brazesti	Cr < Ni	-	-	Cu(6.9)	Zn(12)	Cd(41)	Pb(70), As(1100)
**Geoaccumulation index (I**_**geo**_**)**							
**Impoundment**	**Practically unpolluted (I**_**geo**_ **< 0)**	**Unpolluted to moderately polluted (0 < I**_**geo**_ **≤ 1)**	**Moderately polluted (1 < I**_**geo**_ **≤ 2)**	**Moderately to heavily polluted (2 < I**_**geo**_ **≤ 3)**	**Heavily polluted (3 < I**_**geo**_ **≤ 4)**	**Heavily to extremely polluted (4 < I**_**geo**_ **≤ 5)**	**Extremely polluted (I**_**geo**_ **> 5)**
Saliste	Cr < Ni < Cu	Zn	Pb(2.0), Cd(2.0)	-	-	-	As(5.5)
Gura Rosiei	Cr < Ni < Cu < Zn < Cd	-	Pb(1.9)	-	-	As(4.5)	-
Stefanca	Cr < Ni < Pb	Zn < Cd	As(1.3)	-	Cu(3.9)	-	-
Sesei	Ni < Cr	Zn < Pb	Cd(1.5), As(1.7)	-	-	Cu(4.5)	-
Cutii	Ni < Cr	Zn	Cd(1.4), Cu(1.5)	-	-	Pb(4.1)	As(7.8)
Sartas	Cr < Ni		Cu(1.8), Zn(1.9)	-	Cd(3.4)	Pb(4.4)	As(8.0)
Brazesti	Cr < Ni	Cu	Zn(1.6)	-	Cd(3.4)	Pb(4.2)	As(8.2)
**Contamination factor (C**_**f**_**)**							
**Impoundment**	**Low (C**_**f**_**<1)**	**Moderate (1 < C**_**f**_**≤3)**		**Considerable (3 < C**_**f**_ **≤ 6)**	**Very high (C**_**f**_**>6)**		
Saliste	Ni < Cr < Cu	Zn(2.6)		Pb(5.9)	Cd(6.1), As(67)		
Gura Rosiei	Cr < Ni < Cu < Zn < Cd	-		Pb(5.8)	As(34)		
Stefanca	Cr < Ni	Pb(1.3), Zn(1.4), Cd(2.0)		As(3.7)	Cu(23)		
Sesei	Ni < Cr	Zn(1.5), Pb(1.6)		Cd(4.1), As(4.9)	Cu(34)		
Cutii	Ni < Cr	Zn(2.9)		Cd(4.1), Cu(4.3)	Pb(26), As(321)		
Sartas	Cr < Ni	-		Cu(5.3), Zn(5.6)	Cd(15), Pb(32), As(384)		
Brazesti	Cr < Ni	Cu(2.6)		Zn(4.6)	Cd(16), Pb(28), As(437)		
**Contamination degree (C**_**d**_**)**							
	**Low (C**_**d**_**<8)**	**Moderate (8 < C**_**d**_ **≤ 16)**		**Considerable (16 < C**_**d**_ **≤ 32)**	**Very high (C**_**d**_**>32)**		
Impoundment	**-**	**-**	**-**		Stefanca (32), Gura Rosiei, (42), Sesei (46), Saliste (83), Cutii (360), Sartas (443), Brazesti (488)		

^a^ based on seven typical metals for the studied area.

Tailings of all seven impoundments present a very high contamination degree with hazardous/priority hazardous metals increasing in the order Stefanca < Gura Rosiei < Sesei < Saliste < Cutii < Sartas < Brazesti. The values of contamination factors reveal qualitative differences among tailings impoundments, thus Stefanca and Sesei exhibit very high contamination with Cu, Saliste with As, Gura Rosiei with Cd and As, Cutii with Pb and As, while Sartas and Brazesti with Cd, Pb and As.

### Environmental contamination risk following ARD generation

Detection by X-ray diffraction (XRD) of primary minerals as sulphide and secondary minerals as sulphate indicates that tailings in these impoundments are still reactive exhibiting potential contamination risk through ARD generation. Several features connected to the ARD generation potential for the tailings impoundments under study are presented in Table 5. According to NNP values, tailings from Sartas and Cutii impoundments are undoubted sources of ARD as a result of the high S content (8.5 and 6.9 kg t^-1^) and limited neutralizing capacity of existing calcite. The potential risk of environmental contamination in case of these tailings is confirmed also by the acidic pH values (2.7 and 2.6) of leachate.

**Table 5:** Tailings characteristics linked to ARD generation potential

**Impoundment**	**pH of leachate**	**S**	**NP**^**a**^	**APP**^**b**^	**NNP**^**c**^	**ARD generation potential**
		**kg t**^**-1**^	**kg CaCO**_**3**_**t**^**-1**^	**kg CaCO**_**3**_**t**^**-1**^	**kg CaCO**_**3**_**t**^**-1**^	
Sartas	2.7	8.5	0.0	26.6	−26.6	certain
Cutii	2.6	6.9	0.6	21.6	−21.0	certain
Saliste	2.9	0.7	0.0	2.2	−2.2	uncertain
Gura Rosiei	3.5	0.8	1.25	2.3	−1.1	uncertain
Sesei	8.2	0.5	12.0	1.6	10.4	uncertain
Stefanca	10.5	0.6	18.7	1.9	16.9	uncertain
Brazesti	8.1	1.6	102	5.0	97.0	no

^a^NP - neutralization potential expressed as kg CaCO_3_ t^-1^ tailings; ^b^APP - acid producing potential expressed in calcite consumed for neutralization (kg CaCO_3_ t^-1^) at pH > 6.3; ^c^NNP - net neutralization potential calculated as the difference NP-APP.

The ARD generation in the Cutii and Sartas impoundments is determined by the oxidation of metal sulphides and hydrolysis or/and dissolution of sulphate minerals especially during rainfalls and infiltration of water in tailings, causing a drop of pH in the range of 1.5–3. The pyrite oxidation by atmospheric oxygen takes place according to equation 1 [26,27].

(1) $$\begin{array}{l} {\mathrm{FeS}}_{2}\left(s\right)+15/{4O}_{2}\left(\mathrm{aq}\right)+7/{2\;H}_{2}O\\ \;=\mathrm{Fe}{\left(\mathrm{OH}\right)}_{3}\left(s\right)+{{\mathrm{SO}}_{4}}^{2-}\left(\mathrm{aq}\right)+{4H}^{+}\left(\mathrm{aq}\right) \end{array}$$

Galena and sphalerite oxidation by aqueous ferric iron (equation 2) generates significantly greater quantities of acid than its oxidation by oxygen (equation 3) [28].

(2) $$\begin{array}{l} 2\mathrm{MeS}\left(s\right)+{4\mathrm{Fe}}^{3+}\left(\mathrm{aq}\right)+{3O}_{2}\left(\mathrm{aq}\right)+{2H}_{2}O\\ \;={2\mathrm{Me}}^{2+}\left(\mathrm{aq}\right)+{4\mathrm{Fe}}^{2+}\left(\mathrm{aq}\right)+{{2\mathrm{SO}}_{4}}^{2-}\left(\mathrm{aq}\right)\\ \;+{4H}^{+}\left(\mathrm{aq}\right) \end{array}$$

(3) $$\mathrm{MeS}\left(s\right)+{2O}_{2}\left(\mathrm{aq}\right)={\mathrm{Me}}^{2+}\left(\mathrm{aq}\right)+\left({{\mathrm{SO}}_{4}}^{2-}\right)\left(\mathrm{aq}\right)$$

This process is confirmed by the presence of anglesite (PbSO_4_) identified in traces by XRD analysis. The hydrolysis process suffered by jarosite resulted in post mining oxidation is presented in equation 4 [13,26-28].

(4) $$\begin{array}{l} {\mathrm{KFe}}_{3}{\left({\mathrm{SO}}_{4}\right)}_{2}{\left(\mathrm{OH}\right)}_{6}\left(s\right)=K^{+}\left(\mathrm{aq}\right)+3\mathrm{FeOOH}\left(s\right)\\ \;+{{2\mathrm{SO}}_{4}}^{2-}\left(\mathrm{aq}\right)\\ \;+{3H}^{+}\left(\mathrm{aq}\right) \end{array}$$

The dissolution process of most common secondary minerals (iron sulphate and gypsum) should be also considered as acid source [26,27].

Minerals with neutralization potential identified in tailings were calcite, effective at pH ≥5.7, and aluminosilicates (albite and biotite), reactive at pH ≤ 3.1 [29-31]. Following the neutralization process, the aluminosilicate minerals liberate ions of Al^3+^, Fe^3+^, Mn^2+^, alkaline, earth-alkaline and ions of hazardous/priority hazardous metals from tailing wastes. The K^+^ and Fe^3+^ ions released from the biotite dissolution are further involved in the precipitation of jarosite that occurs in oxidized tailings in acidic environments [31,32]. These processes are most likely to occur in the Sartas impoundment, since the mineralogical analysis revealed a high concentration of biotite and jarosite. The source of Ca^2+^ ions necessary for the formation of gypsum, another secondary mineral associated with ARD generation, is the dissolution of calcite, anorthoclase and feldspar.

Tailings from the Brazesti impoundment does not represent an ARD source as shows the positive NNP determined by a content of 97 kg CaCO_3_ t^-1^ tailings in excess compared with the required stoichiometric amount. The other impoundments were found to be uncertain sources of ARD.

### Environmental contamination risk with hazardous/priority hazardous metals and ions

**Figure 2:**
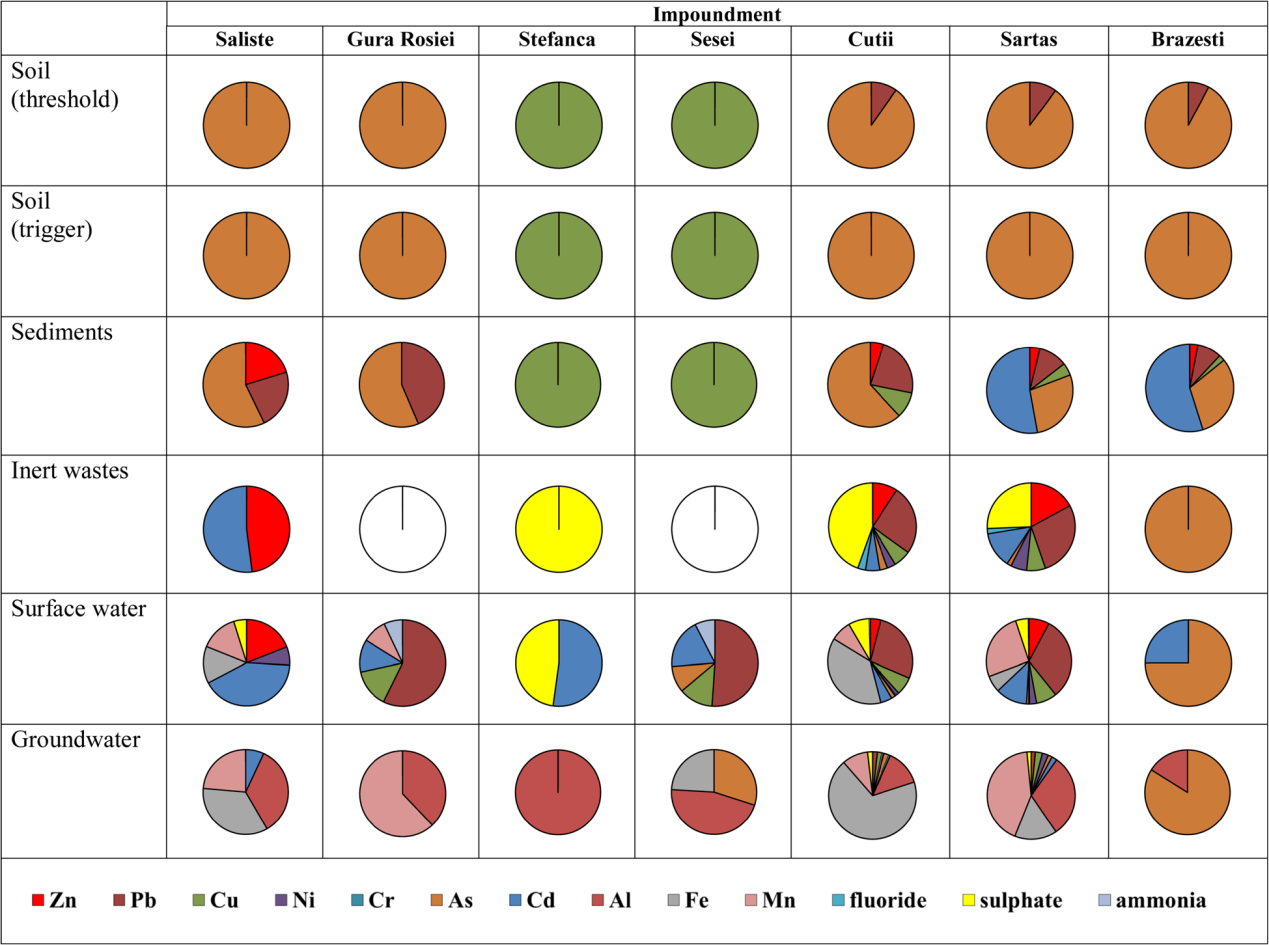
**Weight of contaminants that exceed the corresponding limit value for soil, sediment, waste and water.** Legend: The weight of each contaminant that exceed the corresponding limit value for soil, sediments, inert wastes, surface and ground waters was calculated as the ratio between the found (Table 3) and reference values (Table 6).

To highlight whether these tailings pose a contamination risk, the total and leached fraction of hazardous/priority hazardous metals and ions concentrations in leachate were taken into account. In order to illustrate the contamination risk for soil, sediments, surface and groundwater the weight of each contaminant that exceeded the limit values was calculated as the ratio between the found and reference values set by quality standards. Similar approach was used for the grouping of analysed tailings as inert or non-inert. The obtained results are presented in Figure 2.

The potential contamination risk by diffusion of metals in soil and river sediments in the anthropogenic impact area was estimated by comparing the total content of hazardous/priority hazardous metals with threshold/trigger levels for less sensitive soil use and sediment quality guidelines complying with the Romanian regulation [33,34] (Table 6). Threshold level is the concentration above which the contaminant should generally be regarded as undesirable and possible unacceptable and some forms of remedial action may be necessary. Trigger level indicates the contaminant concentration above which it is considered as posing an environmental risk and clean-up is necessary. The comparison allowed to classify tailings as follows: unpolluted (group 1) when the total metal content was below the threshold level; medium polluted (group 2) for contents between threshold and trigger levels and highly polluted (group 3) for values exceeding trigger levels. Based on these considerations tailings from all impoundments were classified as belonging to group 3 of high contamination risk for soil, those of Saliste, Gura Rosiei, Cutii, Sartas and Brazesti because of As, while those of Stefanca and Sesei because of Cu. In terms of Pb, the impoundments Cutii, Sartas and Brazesti were found to correspond to group 2 of medium contamination risk. Tailings were also found to present a high contamination risk for sediment because the total content of hazardous/priority hazardous metals exceeded the sediment quality guidelines. The contamination risk of sediment decreases in the order: Brazesti≈Sartas>Cutii (Cd>Pb>Cu>Zn)>Saliste>Gura Rosiei (As>Pb)>Stefanca≈Sesei (Cu).

**Table 6:** Limit values according to quality guidelines for inert waste, soil, sediment and water

**Parameter**	**Leaching limit values for inert waste**[35]	**Soil guidelines for less sensitive use**[33]		**Sediment quality guideline**[34]	**Surface water quality standards**^**a**^[34]	**Threshold value for groundwater**[37]
		**Threshold**	**Trigger**			
	**mg kg**^**-1**^	**mg kg**^**-1**^	**mg kg**^**-1**^	**mg kg**^**-1**^	**mg l**^**-1**^	**mg l**^**-1**^
Al	-	-	-	-	-	0.2^b^
Fe	-	-	-	-	0.3–2	0.2^b^
Mn	-	2000	4000	-	0.05–1	0.05^b^
Ba	20	1000	2000	-	-	-
Zn	4	700	1500	150	0.1–1	5^b^
Pb	0.5	250	1000	85	0.005–0.05	0.01^b^
Cu	2	250	500	40	0.02–0.1	0.1^b^
Ni	0.4	200	500	35	0.01–0.1	0.02^b^
Cr	0.5	300	600	100	0.025–0.25	0.05^b^
As	0.5	25	50	29	0.01–0.1	0.01^b^
Cd	0.04	5	10	0.8	0.0005–0.005	0.005^b^
Co	-	100	250	-	-	-
V	-	20	40	-	-	-
Ag	-	200	400	-	-	-
F-	10	-	-	-	-	-
Cl^-^	800	-	-	-	25–300	250
SO_4_^2-^	1000	-	-	-	60–300	310
NO_3_^+^	-	-	-	-	1–11.2	50^b^
NH_4_^+^	-	-	-	-	0.4–3.2	0.7

^a^ Minimum value-very good ecological status; maximum value-bad ecological status;

^b^MAC (Maximum Allowable Concentrations) for drinking water [40].

Beside the total content of metals, another useful criterion to assess the potential risk of environmental contamination used in this study was the leaching fraction of metals and ions because it expresses water mobility. The reason is that even low total concentrations of a contaminant can exhibit high leachability and thus a significant risk for environment and vice versa [12]. Comparison of the leachable fraction of metals and ions in tailings at a solid-to-water ratio of 1:10 (Table 3) with the leaching limit values for inert waste [35] (Table 6) shows that the requirements are fulfilled only for the Gura Rosiei and Sesei impoundments, while the other tailings cannot be considered of no contamination risk. The highest exceeding of leaching limit value for inert waste was registered for the tailings of Sartas (Zn, Cu, Ni, F^-^, SO_4_^2-^) and Cutii (Zn, Cu, SO_4_^2-^) impoundments (Figure 2). In fact, the Acid Base Accounting (ABA) test indicated these tailings as considerable sources of ARD, which explained the high concentration of the previously mentioned elements and anions in leachate. The accepting criteria for inert wastes of some indicatives were also surpassed in the tailings leachates of Saliste (Zn, Cd), Stefanca (SO_4_^2-^) and Brazesti (As).

To highlight the possible contamination risk for surface water, content of metals and ions in leachate were compared with limit values in accordance with [34]. The contamination risk for groundwater was also investigated. The European Community has prepared Guidelines for the establishment of own threshold values of pollutants in groundwater by Member States [36] considering the lithological and hydrogeological characteristics of the groundwater bodies including information on background levels and water balance. According to [37], in the case of the Aries basin there were established threshold values for groundwater only in terms of Cl^-^, SO_4_^2-^ and NH_4_^+^. Based on the 2000/60/EC Directive [38] and a case study [39], the threshold values for metals in groundwater in Romania were establish to be the Maximum Allowable Concentrations (MAC) in drinking water [40]. The exceeding of the limit values for surface and groundwater by the considered contaminants in leachate reveals significant differences in risk contamination from one impoundment to another. According to quality standards for surface water, leachates generated by tailings from the Sartas and Cutii impoundments were found to have bad ecological status (V) and thus the highest contamination risk because of Fe, Cu, Zn, Cd, Ni and SO_4_^2-^ concentration. Poor status (IV) was attributed to leachate generated by tailings of the Saliste (Fe, Cu, Cd) and Brazesti (As) impoundments, and moderate ecological status (III) to leachate from Sesei (Cu, Cd) and Gura Rosiei and Stefanca (Cd). The level of sulphate in leachate is mainly controlled by the solubilisation of gypsum, jarosite and ferric sulphate, while that of trace metals by the desorption from aluminosilicates and S-containing acid waste rocks. To sum up, tailings from impoundments represent a contamination risk for the Aries river basin in wet seasons according to the classification of the corresponding leachates. The groundwater contamination risk by Al, As, Cd, Cu, Fe, Mn, Ni, Pb and SO_4_^2-^ could come mainly from the Cutii and Sartas impoundments. Contamination by As could also originate from tailings stored in the Sesei and Brazesti impoundments, while by Al, Cd, Fe and Mn from Saliste.

### Principal component analysis and agglomerative hierarchical clustering

Multivariate statistical analysis for evaluation and interpretation of the data allows discriminating between natural and anthropogenic origin of contaminants and pollution levels respectively, within a contaminated site [41-43]. R-mode PCA was applied to total content of metals and sulphur in tailings and leached fractions of metals and ions. According to the Kaiser criterion, only the PC’s with eigenvalue higher than 1.0 was retained and subjected to varimax rotation. Factor loadings used to determine the relative importance of a variable as compared to other variables in a PC were classified as ‘strong’, ‘moderate’, and ‘weak’ corresponding to absolute loading value of >0.75, 0.50–0.75, and 0.30–0.50 respectively [44,45].

The varimax rotated loadings of 4 PC’s with eigenvalues >1 (95% of the system variability) considering as variables the total element contents and ABA test parameters are presented in Table 7. Data in Table 7 show that hazardous/priority hazardous metals exhibit different association to waste rocks. The first factor (PC1) accounting for 47% of the total variance was attributed to alkaline silicate rocks (aluminosilicates and ferrosilicates) of low neutralization potential and host for Cu, Co, V and Ti mineralization. The lack of correlation between S and Ba in this PC shows that Ba is not present as barite (BaSO_4_) of anthropogenic origin. In turn, the positive correlation with biogenic elements (K, Na) suggests the biogenic origin of Ba that can be retained by adsorption on aluminosilicates or iron oxides following diagenetic processes. Barium has the ability to replace biogenic elements like K in sedimentary rocks due to their similar ionic radius [46]. The negative loading factors of Au and Ag in this PC indicate that precious metals are not associated with silicates and exhibit different mineralization type. Low loadings of Au and Ag in the other three PCs show that precious metals have no significant influence in describing variability of tailings composition.

**Table 7:** Loadings of the 4 PC’s considering as variables the total metals, S and ABA parameters

**Parameter**	**PC1**	**PC2**	**PC3**	**PC4**
Al	0.868**	−0.248	−0.082	0.108
Fe	0.551*	0.618*	−0.048	0.096
Ca	0.000	0.904**	0.191	−0.015
K	0.797**	−0.002	−0.218	0.152
Mg	0.862**	−0.199	0.207	0.033
Na	0.869**	−0.208	−0.119	−0.027
Mn	0.836**	0.009	0.329	0.182
Ba	0.662*	−0.154	−0.196	0.518*
Zn	−0.134	0.743*	0.176	0.495
Pb	−0.273	0.872**	0.100	0.101
Cu	0.862**	−0.239	−0.095	0.001
Ni	0.060	0.613*	−0.047	0.640*
Cr	0.220	0.575*	−0.127	0.659*
As	−0.247	0.843**	0.273	0.095
Cd	−0.124	0.612*	0.421	0.533*
Co	0.855**	−0.169	0.126	0.093
Sr	0.820**	0.339	0.210	−0.043
Ti	0.856**	−0.337	−0.088	0.005
V	0.739*	−0.428	−0.092	0.044
W	−0.220	0.858**	0.245	0.015
Ag	−0.850**	−0.163	−0.292	0.145
Au	−0.811**	0.035	−0.218	0.161
NNP	0.122	0.002	0.915**	−0.023
S	−0.149	0.799**	−0.432	0.087
NP	0.093	0.232	0.890**	0.000
APP	−0.145	0.800**	−0.432	0.089
Variability (%)	47	31	12	5

**strong influence on the latent factor (>0.75); *moderate influence on the latent factor (0.50-0.75).

The second principal component (PC2) with a contribution of 31%, attesting association of S with Fe, was attributed to S-containing minerals (sulphides and sulphates), which host Zn, Pb, Cd, As and W. These minerals constitute the main contamination source of soil and water in surrounding areas with hazardous (As, Zn) and priority hazardous (Cd, Pb) metals resulted from sulphide oxidation and dissolution of soluble sulphates.

PC3 (12%) was attributed to carbonate minerals of high ARD neutralization capacity. The positive correlation between NP and NNP indicates sufficient neutralization potential of tailings with two exceptions. It was previously shown that the tailings impoundments of Cutii and Sartas are certain sources of ARD and thereby Cd and Mn can be released from carbonate host rocks on which these elements are preferentially retained. However, the low factor loadings of Cd and Zn, Pb, As in PC3 compared to PC2 show that carbonates are not the main source of hazardous metals in water.

Chromium and Ni with low influence (5%) on the variability of elemental composition of tailings are included in PC4 ascribed to traces of biogenic elements. The correlation between Cr, Ni and Ba, although was moderate, it suggests the biogenic origin of these two metals, as a result of their adsorption in clay minerals by diagenetic processes [46].

PCA analysis on data obtained from the leaching study shows that 96% of variability is shared among four PC’s (Table 8). The first component (PC1) accounting for 61% variability was associated to priority hazardous (Cd, Ni) and hazardous (Cu, Co, Cr, Zn) metals as well as Mn and anions (F^-^, SO_4_^2-^) retained on Al and Mg minerals leached in water. In accordance with its factor loading, sulphate has a moderate influence on the metals mobility in water. PC2 (18%) was attributed to biogenic elements (K, Na, Cl^-^, NO_3_^-^) with no contamination risk for water. The third component (PC3) of 11% variability was assigned to priority hazardous (Pb) and hazardous metals (Cr) as well as ions (F^-^, SO_4_^2-^, NH_4_^+^) leached from oxidized S-containing minerals (Fe and Ca sulphates). The last component (PC4) explaining 6% of variability was associated to biogenic matter. Although moderate, the correlation between dissolved organic carbon (DOC) and water leachable Ba fraction suggests the biogenic origin of this element in tailings. This finding is in accordance with the literature data regarding the effect of organic matter on Ba availability [47,48]. Although in the environment As has a high affinity for the organic matter, in the studied tailings this component has a low influence on the mobilization of As and other hazardous/priority hazardous metals by water [49]. This behaviour is explained by the relatively low content of organic matter (0.5–2.3 kg t^-1^) in the studied tailings. Also, increasing pH favoured especially the mobilization of biogenic elements linked to organic matter as shown by the positive associations in PC2 and PC4.

**Table 8:** Loadings of the 4 PC’s considering as variables the leached metals and ions concentration

**Parameter**	**PC1**	**PC2**	**PC3**	**PC4**
Al	0.864**	−0.017	0.330	0.019
Fe	0.070	−0.143	0.909**	0.065
Ca	0.278	−0.047	0.875**	0.092
K	−0.105	0.873**	−0.193	0.211
Mg	0.903**	−0.109	0.061	0.087
Na	−0.097	0.914**	−0.053	0.078
Mn	0.905**	−0.059	0.181	−0.001
Ba	0.083	0.682*	−0.031	0.513*
Zn	0.874**	−0.095	0.287	−0.015
Pb	0.589*	−0.178	0.685*	0.077
Cu	0.792**	−0.109	0.465	0.032
Ni	0.882**	−0.083	0.267	−0.005
Cr	0.566*	−0.280	0.612*	−0.011
As	0.370	−0.362	0.400	0.413
Cd	0.901**	−0.085	0.188	−0.027
Co	0.900**	−0.064	0.202	−0.004
Sr	0.322	0.277	0.802**	0.153
Ti	0.584*	−0.130	0.696*	0.104
pH	−0.319	0.543*	−0.411	0.516*
EC	0.543*	−0.048	0.745*	0.045
DOC	0.073	0.069	0.398	0.791**
F^-^	0.653*	−0.027	0.649*	0.082
Cl^-^	−0.008	0.734*	0.459	−0.170
NO_3_^-^	−0.189	0.852**	−0.120	−0.227
SO_4_^2-^	0.579*	−0.088	0.715*	0.055
NH_4_^+^	0.207	−0.152	0.664*	−0.290
Variability (%)	61	18	11	6

**strong influence on the latent factor (>0.75); *moderate influence on the latent factor (0.50–0.75).

**Figure 3:**
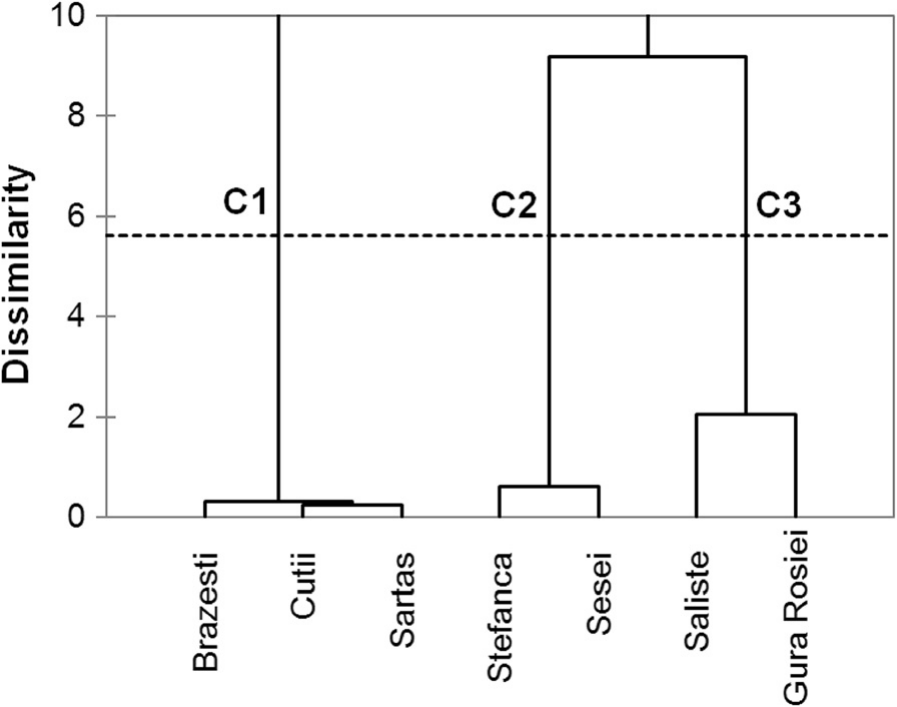
Dendrogram showing the clustering of tailings based on elemental composition and ABA parameters.

The AHC on element concentrations in aqua regia extracts of tailings and neutralization parameters resulted from the ABA test considering tailings impoundments as objects is presented in Figure 3. The grouping of tailings in 3 clusters is consistent with the contamination degree of the stored waste. Cluster C1 groups the tailings of Brazesti, Sartas and Cutii impoundments exhibiting the highest contamination with As, Pb and Cd, while the other two clusters include tailings with lower contamination and thus lower environmental risk, namely Sesei and Stefanca (C2) and Gura Rosiei and Saliste (C3). The grouping of these four impoundments in two sub-clusters is due to the difference in respect with the main contaminants, namely Cu (Sesei and Stefanca) and As and Cd (Gura Rosiei, Saliste), respectively.

**Figure 4:**
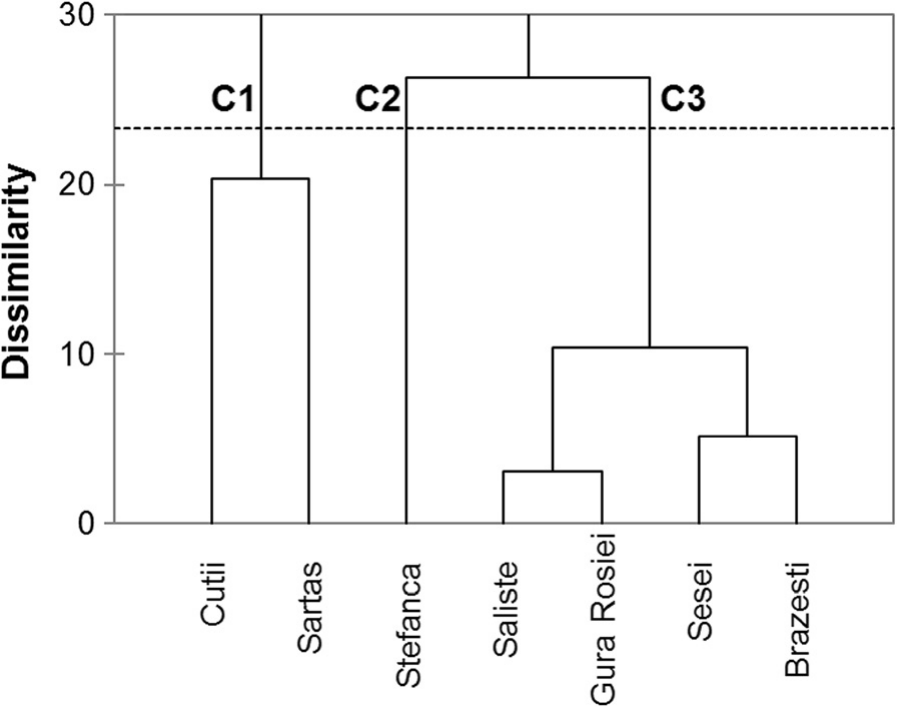
Dendrogram showing the clustering of tailings based on leachate composition.

The dendrogram produced by clustering tailings considering metals and ions concentrations, pH and electrical conductivity in leachate is presented in Figure 4. Tailings impoundments are grouped in three clusters according to their ARD generation potential. Thus cluster C1 includes the tailings of Cutii and Sartas impoundments not complying with inert waste criteria. Clusters C2 and C3 group tailings of lower contamination risk for water and classified as uncertain or low ARD generation potential.

## Conclusions

Tailings from mineral processing of Cu porphyry deposits and stored in seven impoundments in the Aries basin, Apuseni Mountains, Romania, were characterized by mineralogical and elemental composition, potential of ARD generation and leaching test of hazardous/priority hazardous metals and ions. The main contributions of our study are:

i. Classification of tailings from this area based on the potential contamination risk for soil, sediments, surface and ground-waters according to reference quality guidelines

ii. Selection of representative hazardous/priority hazardous metals for the studied impoundments, that allowed to classify the tailings by contamination indices and to highlight the differences between the impoundments related to contaminants influence on the environmental quality

iii. Interpretation of results using multivariate statistics allowed to distinguish between the natural and anthropogenic origin of metals and their grouping in 4 principal components that describe the tailings elemental composition and water leachable fraction of contaminants

iv. Grouping of the tailings impoundments by AHC considering their contamination and ARD generation potential.

Results have indicated that tailings exhibit very high contamination degree for soil, especially with As, Pb, Cu and Cd. Based on the total contents of these elements, three impoundments were found to represent high and medium contamination risk with As, Pb and Cd, whereas two impoundments show high contamination risk with As and other two with Cu. The tailings impoundments evidenced a high potential contamination risk with As, Pb, Cu and Cd of river sediment. Two tailings impoundments (Cutii and Sartas) were classified as hazardous because of the leached fraction of metals and ions and ARD generation potential. Groundwater contamination with Fe, Al and Mn is also possible.

Principal component analysis has shown that the variability of total element contents and that of leachable hazardous/priority hazardous metals and ions are controlled mainly by alkaline silicates containing Al and Mg, and acidic minerals with S, Fe and Ca. Organic matter had low influence on the element leachability excepting some biogenic elements such as Ba. Increasing pH favours only the mobilization of biogenic elements of no contamination risk. Although, PCA offers important information concerning the influence of different parameters on the hazardous/priority hazardous metals behaviour in tailings, has also limitations and disadvantages. The main limitations are that results interpretation in not based on the original data but on a new set of independent variables (PCs) and the resulting PCs have limited comparability among different studies. The reduction of data set is based on linear transformations and the maximisation of PCs is made in the detriment of others with less variability. In addition, it requires large dataset but is difficult to deal with missing data or outliers.

Although some measures have been taken to green the tailings impoundments, they continue to act as potential contamination sources of the environment and are vulnerable by rainwater infiltration to mobilization of hazardous/priority hazardous metals and ions from secondary minerals. In our opinion, a monitoring program of these tailings impoundments would be helpful to continue, even after greening completion.

## Methods

### Collection and preparation of samples and analytical methods

Five sub-samples from each of the seven tailings impoundments were randomly collected in July 2012 from 25 cm depth using a polypropylene shovel. For each impoundment the sub-samples were mixed to obtain a composite sample that was subjected to analysis. The bulk mineralogical composition was determined by powder X-ray diffraction (XRD) using the high-resolution Bruker D8 Advance diffractometer (Bruker-AXS, Karlsruhe, Germany) at Cu K_α_ radiation after grinding and homogenizing of samples to <63 μm. Samples were analysed for total metal concentrations by inductively coupled plasma atomic emission spectrometry (ICP-AES) and inductively coupled plasma mass spectrometry (ICP-MS) after mineralization. An amount of 1 g dried sample (105±5 °C) was treated with 28 ml aqua regia according to SR ISO 11466:1995. The filtrate was diluted to 100 ml with ultrapure water and the contents of Ca, Mg, Na, K, Al, Fe, Cu, Pb, Zn, Mn, Ba and Sr were determined by ICP-AES (OPTIMA 5300 DV, Perkin Elmer, Norwalk, USA), while those of Cd, As, Ti, Ni, Cr, Co, Au, Ag, V, W by ICP-MS (ELAN DRC II, SCIEX, Perkin Elmer, Toronto, Canada). Arsenic was determined as ^75^As^16^O^+^ polyatomic ion using the dynamic reaction cell technology at 0.8 ml min^-1^ oxygen and RPq = 0.45. The ICP-AES technique was previously validated for the analysis of granular waste with metal recovery in the range 91–109% and 0.8–4.6% repeatability [50]. Sulphur content in tailings samples was determined by X ray fluorescence using the spectrometer α6500 (INNOV-X, Woburn, MA, US).

To characterize the water mobility of hazardous/priority hazardous metals and ions, tailings were subjected to leaching at a solid-to-water ratio of 1:10 as described in SR EN 12457–2:2003. An amount of wet granular sample with particle size below 4 mm corresponding to 90 g dry mass was leached for 24±0.5 h at room temperature (20±5 °C) using the REAX 20 overhead shaker (Heildolph, Schwabach, Germany). The leachate was analysed for metal contents by ICP-MS and main anions (Cl^-^, NO_3_^-^, F^-^, SO_4_^2-^) respectively, by ion-liquid chromatography according to ISO 10304–1:2007 using the 761 Compact IC (Metrohm, Herisau, Switzerland).

Ammonium ions in leachate were determined as indophenol blue complex according to SR ISO 7150–1:2001 using the spectrophotometer Lambda 25 (Perkin Elmer, Beaconsfield, UK). The electric conductivity and pH of leachate were measured with the 350i multiparameter (WTW, Wilheim, Germany), while the DOC content was assessed according to SR EN 1484:2006 using the Multi N/C 2100S Analyser (Analytik Jena, Jena, Germany).

The ABA test was conducted to evaluate the ARD generation capacity of tailings estimated by NNP. According to Sobeck et al. [51] it is calculated as the difference between Neutralization Potential (NP) and Acid Producing Potential (APP) assuming that sulphur is present only in the form of pyrite, while carbonate as calcite (CaCO_3_). The NP expressed as the content of CaCO_3_ (kg t^-1^) resulted from the titration with NaOH solution, of the acid excess used for sample mineralization. The APP level at pH < 6.3 was estimated considering that each S percent requires for neutralization 31.25 kg CaCO_3_ t^-1^ tailings. The material is considered as ARD generating when the NNP value is lower than −20 kg CaCO_3_ t^-1^, while a value higher than +20 kg CaCO_3_ t^-1^ indicates that the material is non-ARD generating. For NNP values between −20 and +20 kg CaCO_3_ t^-1^ the prediction about the ARD generation is uncertain [52,53].

### Statistical analysis

The XLStat Microsoft Excel plug-in (Addinsoft) was used for the statistical processing of the data. Principal Component Analysis with varimax rotation was used to interpret the structure of the main dataset. Agglomerative Hierarchical Clustering using the Ward’s linkage method and Euclidian distances as a measure of similarity was used to group the determined parameters into classes.

## Competing interests

The authors declare that they have no competing interests.

## Authors’ contributions

EL conceived the study, collected the samples, performed S and DOC determinations, leaching and ABA tests, statistical analysis and helped to manuscript preparation. TF interpreted the results and coordinated the manuscript writing. MP participated to the interpretation of the results and to the manuscript preparation. CT determined the elemental composition of samples and participated to data analysis. GB determined the mineralogical composition of samples by XRD analysis and data interpretation. All authors read and approved the final manuscript.
